# Biofilm-forming traits enrich the plasmid diversity and functional potential in particle-attached bacteria in coastal ecosystems

**DOI:** 10.1128/spectrum.00460-26

**Published:** 2026-06-15

**Authors:** Zhendu Mao, Mengying Jiang, Zifan Zhao, Shumin Xu, Heng Wang, Kelin Chen, Jianglang Duan, Zhuo Chen, Dan He, Peng Xing, Qinglong L. Wu

**Affiliations:** 1 Center for Evolution and Conservation Biology, Southern Marine Sciences and Engineering Guangdong Laboratory (Guangzhou), Guangzhou, China; 2 Department of Ocean Science and Engineering, Southern University of Science and Technology 255310, Shenzhen, China; 3 School of Life Sciences, Institute of Life Science and Green Development, Hebei University 56667, Baoding, China; 4 Department of Ecology and Institute of Hydrobiology, Jinan University 47885, Guangzhou, China; 5 School of Environmental Science & Engineering, Guangzhou University 47875, Guangzhou, China; 6 Key Laboratory of Lake and Watershed Science for Water Security, Nanjing Institute of Geography and Limnology, Chinese Academy of Sciences 66289 , Nanjing, China; 7 Sino-Danish Center for Science and Education, University of Chinese Academy of Sciences 74519, Beijing, China; 8 Lake Fuxian Ecological Research Station, Chinese Academy of Sciences, Chengjiang, China; Connecticut Agricultural Experiment Station, New Haven, Connecticut, USA

**Keywords:** coastal ecosystems, particle-attached, free-living, plasmidome, resistome, biofilm-forming traits

## Abstract

Planktonic microorganisms play a central role in aquatic biogeochemical processes and are commonly divided into particle-attached (PA) and free-living (FL) fractions. Although these two lifestyles differ in ecological strategy, the contribution of plasmids to their niche differentiation remains poorly resolved. Here, we conducted a plasmid-centric metagenomic analysis of two anthropogenically impacted coastal ecosystems in South China, the Pearl River Estuary (PRE), and Daya Bay (DYB), to determine the environmental and biological drivers of plasmid diversity, and their functional potenitial. We found that plasmid diversity was jointly shaped by different fractions and environmental stressors. The PA fraction contained significantly higher plasmid abundance and richness than the FL fraction, and was enriched in multifunctional and conjugative plasmids. These plasmids were associated with genes adapting to the PA lifestyle or microenvironments, suggesting linkage between particle attachment and plasmid maintenance. Structural equation modeling indicated that different fractions shaped plasmid diversity primarily through biofilm-forming genes. Along an anthropogenic gradient from DYB to PRE, increasing pollution levels were accompanied by higher plasmid diversity and greater abundances of antibiotic and metal resistance genes. Plasmid diversity was strongly correlated with resistance gene abundance. The enrichment of transferable plasmids in the PA fraction, where cell densities are high and intercellular distances are close, suggested that particle-associated habitats favor genetic exchange and the persistence of resistance traits. Together, these results demonstrate that particle-associated microbial communities represent key reservoirs of plasmid diversity and resistance potential in coastal ecosystems and highlight the combined influence of lifestyles and anthropogenic stress on plasmid-mediated microbial adaptation.

**IMPORTANCE:**

Plasmids play an important role in microbial adaptation by mediating horizontal gene transfer, yet the ecological contexts that favor their persistence and diversification in natural environments remain poorly understood. This study showed that particle-attached microbial communities in coastal waters harbored substantially higher plasmid diversity and resistance potential than free-living communities, and that this enrichment is strongly linked to biofilm-associated traits. By demonstrating how particulate habitats and pollution gradients jointly shape plasmid diversity and resistance gene abundance, our findings identify particle-associated microenvironments as critical reservoirs for plasmid-mediated functions in coastal ecosystems. These results advance understanding of how microbial lifestyle and human activities influence microbial evolution and the environmental dissemination of resistance traits.

## INTRODUCTION

Planktonic microorganisms play a fundamental role in aquatic ecosystems, mediating the transformation of nutrients and contaminants, and sustaining ecosystem health (1). These microorganisms are typically partitioned into two distinct size-based ecological fractions (hereinafter called size fractions): particle-attached (PA) and free-living (FL) (2). Despite their spatial proximity, their lifestyles represent markedly different ecological strategies (3). Consequently, PA and FL microorganisms often exhibit pronounced divergence in taxonomic composition and metabolic potential (4–6). Elucidating the mechanisms that generate and maintain functional divergence between PA and FL communities is therefore central to microbial ecology and to understanding microbial adaptation and biogeochemical regulation in aquatic systems.

Plasmids are extrachromosomal, autonomously replicating genetic elements and are pivotal drivers of microbial ecology and evolution (7). Although not essential for the host survival at any condition, plasmids frequently confer fitness advantages in specific niches by providing genes for antibiotic resistance, heavy metal tolerance, and specialized metabolic pathways (8–10). Furthermore, plasmids serve as scaffolds for other mobile genetic elements (MGEs), such as insertion sequences and transposons, which facilitate the integration of beneficial traits into host chromosomes and accelerate evolutionary adaptation (7, 11). Accompanied by these benefits, plasmid maintenance imposes a metabolic burden on the host (12), and its persistence depends on the net fitness gain provided under prevailing environmental conditions (9). Previous studies have focused primarily on the mechanisms by which conjugative plasmids persist, disperse, and interact with host cells (7, 8, 13, 14). A comprehensive understanding of how plasmid diversity and its associated functions are distributed across different ecological fractions remains unclear.

Aquatic microbial communities, separating into PA and FL fractions, offer an ideal model system to investigate plasmid distribution patterns. From a biophysical perspective, the high microbial density and physical proximity within the PA fraction facilitate the horizontal gene transfer (HGT) required for conjugation (15). Thus, mobilizable/conjugative plasmids may benefit from this physical structure. From a genomic perspective, PA microorganisms typically possess larger genomes than FL microorganisms (16, 17); given that plasmids contribute to total genomic content, the larger genome of PA microbes may benefit from owning more and larger plasmids. Furthermore, previous studies have reported an enrichment of antibiotic resistance genes (ARGs) and/or metal resistance genes (MRGs) in PA fractions (18–22). Besides, as plasmids are important vectors for resistance genes, they may play an important role in shaping the distinct resistome profiles of PA and FL microbial communities (23). Therefore, exploring the distribution of plasmid diversity in PA and FL fractions will not only help us understand how ecological factors affect plasmid diversity, but also how plasmids contribute to the functional differences of PA and FL patterns reported in previous studies.

Plasmid persistence and diversity are influenced by a complex interplay of host taxonomy, plasmid–host compatibility, and environmental stressors (7, 15, 24). A recent study suggests that biofilm-forming traits strongly contribute to higher plasmid diversity and larger plasmid sizes at the strain level (25). Some PA microenvironments can recruit microorganisms with biofilm-forming traits, like algae-associated environments (26, 27). Thus, different diversity of plasmid between PA and FL fractions may be driven by biofilm-forming traits at the community level. Comparing their contributions to shaping plasmid diversity in aquatic ecosystems could help us understand the pattern of plasmid biogeography.

In this study, we hypothesize that (i) due to the biophysical traits of PA environments, PA microbial communities harbor higher diversity and functional potential of plasmids than FL communities, and this distribution was likely shaped by both higher host diversity and enrichment of bacteria with biofilm-forming traits in the PA fraction; (ii) when considering the spatial distribution of plasmids, they are affected by both size fractions, that is, different niches, and environmental gradients, the latter being primarily represented by human activities; (iii) as an important vector for resistance genes, the abundance of plasmids may closely relate to the accumulation of ARGs and MRGs, further leading to higher abundance of ARGs and MRGs in the PA fraction. To test these hypotheses, we conducted a plasmid-centric metagenomic analysis in two anthropogenically influenced coastal ecosystems (Fig. S1): the Pearl River Estuary (PRE) and the Daya Bay (DYB). These two regions serve as critical models due to their significant exposure to anthropogenic pollutants, including antibiotics and heavy metals (28–30). By integrating plasmid diversity with functional annotation and environmental parameters, this study aims to elucidate the ecological mechanisms governing plasmid dynamics and to clarify their role in structuring microbial functions across contrasting ecological fractions.

## RESULTS

### Environmental variables of PRE and DYB

**Fig 1:**
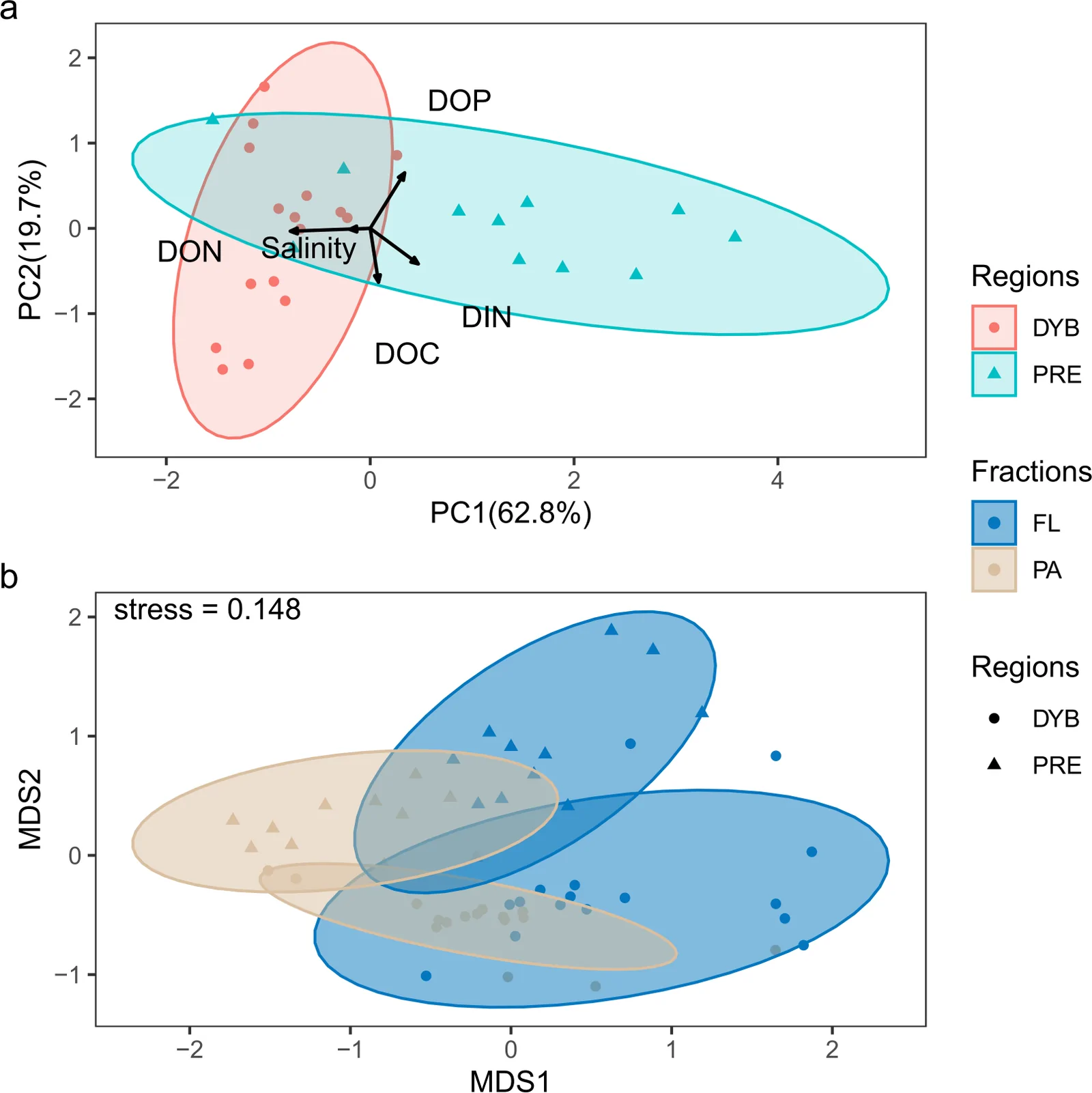
Overview of environmental variables and bacterial community differences across different particle size fractions in Daya Bay (DYB) and the Pearl River Estuary (PRE). (**a**) Principal component analysis of environmental parameters reveals differences between the sampling regions of DYB and PRE. Concentrations of dissolved inorganic nitrogen (DIN), dissolved organic carbon (DOC), dissolved organic phosphorus (DOP), and dissolved organic nitrogen (DON) were first log-transformed and further analyzed. Only environmental parameters with any axis larger than 0.1 were reserved. PC1 and PC2 explain 62.1% and 19.5% of the variance, respectively. (**b**) Nonmetric multidimensional scaling based on Bray-Curtis’s dissimilarity on bacterial community structure, showing the community differences between regions (DYB vs PRE) and fractions (particle-attached, PA vs free-living, FL). Confidence ellipses indicate the 95% confidence interval.

Water samples were collected from 27 sites, including 16 from DYB and 11 from PRE (Fig. S1). Significant differences in environmental variables were observed between PRE and DYB (Multiple response permutation procedures: *P* = 0.001). PCA revealed that dissolved organic nitrogen (DON), dissolved inorganic nitrogen (DIN), dissolved organic phosphorus (DOP), and salinity were the primary drivers of environmental differences, as their contribution to the first principal component (PC1) larger than 0.1; PRE exhibited higher concentrations of DIN and DOP, whereas DYB was characterized by higher salinity and DON (Fig. 1a). Furthermore, analysis of historical data (2017–2025) for DYB and PRE showed that PRE experienced more severe pollution than DYB, with higher levels of petroleum hydrocarbons and chemical oxygen demand and more frequent occurrences of “worse than Class IV” water quality grades (Fig. S2). These results indicated that PRE was subject to stronger anthropogenic pressure than DYB.

### Enriched bacterial diversity in the PA fraction

As all predicted plasmid hosts were bacteria, we focused on bacterial communities rather than total prokaryotic communities based on 16S rRNA gene amplicon sequencing. PA communities exhibited greater numbers of observed amplicon sequence variants (ASVs), as well as greater numbers of observed genera and families (Fig. S3a through c). Bacterial community composition differed significantly between PA and FL fractions (ANOSIM: *P* < 0.001; Fig. 1b). The PA fraction was dominated by Betaproteobacteria (21.4%), Cyanobacteriota (17.4%), and Bacteroidota (14.0%), whereas the FL fraction was dominated by Alphaproteobacteria (20.9%), Gammaproteobacteria (15.2%), and Bacteroidota (11.9%) (Fig. S3d).

### Plasmid diversity and functional potential in PA and FL fractions

**Fig 2:**
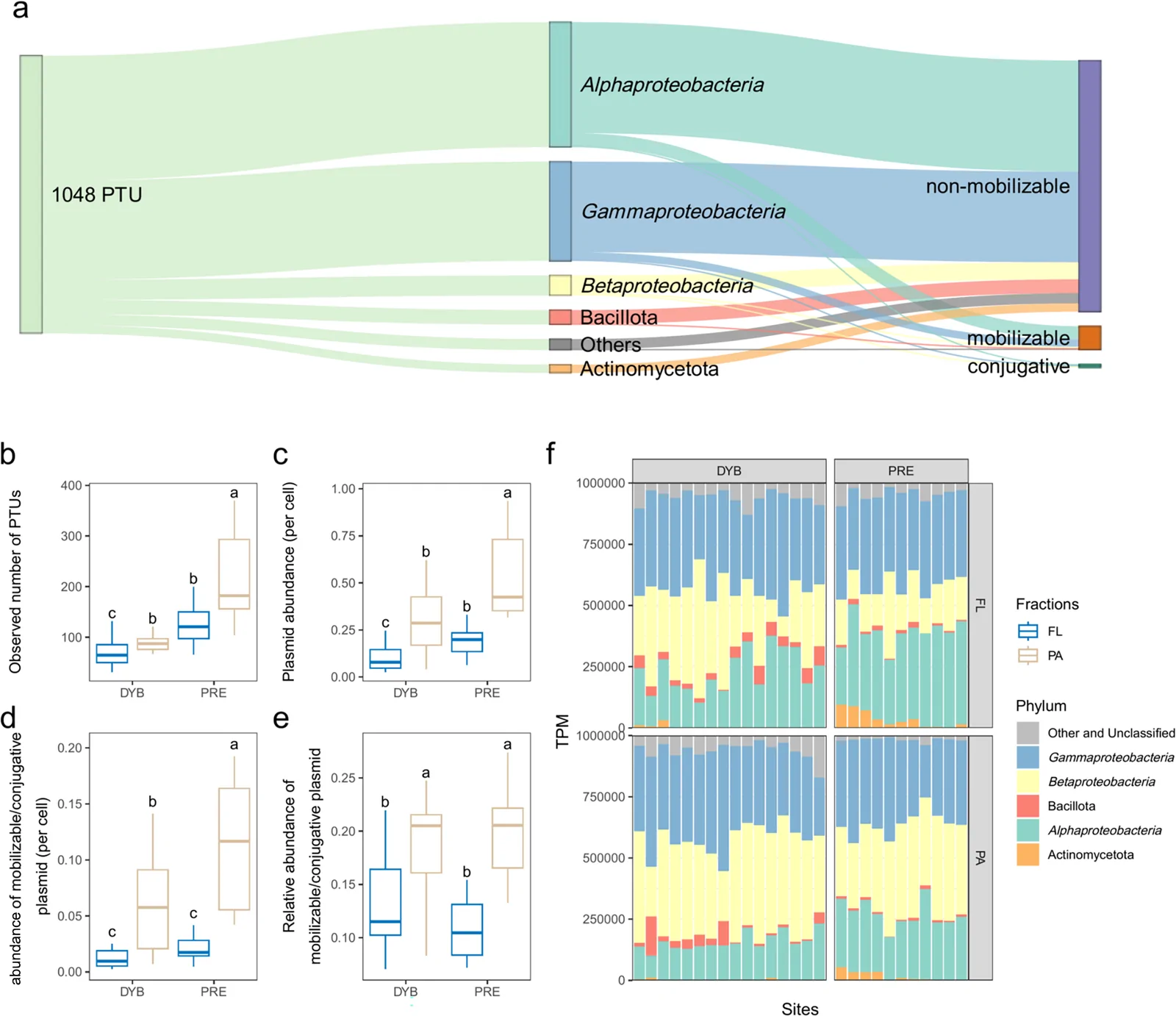
Distribution, abundance, and host characteristics of plasmids in different size fractions from DYB and PRE. (**a**) Taxonomic distribution at the phylum level of hosts for the 1,048 plasmid taxonomic units (PTUs) and their predicted mobility (categorized as conjugative, mobilizable, or non-mobilizable). (**b–e**) Box plots showing comparisons between regions and size fractions (PA: particle-attached; FL: free-living): (**b**) number of observed PTUs; (**c**) absolute plasmid abundance (mean plasmid copies per cell, estimated by normalization with single-copy marker genes); (**d**) absolute abundance of mobilizable and conjugative plasmids; and (**e**) relative abundance of mobilizable and conjugative plasmids (proportion of total plasmid abundance). (**f**) Relative abundance of plasmids categorized by the predicted phylum of their hosts for different samples. Relative abundance was normalized using the TPM method. The phylum Pseudomonadota was separated into *Alphaproteobacteria*, *Gammaproteobacteria*, and *Betaproteobacteria*.

A total of 3,881 plasmids were recovered from 54 metagenomic samples and clustered into 1,046 PTUs with 95% nucleotide identity and 80% coverage. Most PTUs were non-mobilizable (947), with smaller proportions identified as mobilizable (88) or conjugative (11) (Fig. 2a). Rarefaction curves indicated that sequencing depth was sufficient to capture most PTUs in the majority of samples (Fig. S4). The PA fraction harbored more PTUs than FL fractions (Wilcoxon test: *P* < 0.001; Fig. 2b). After normalizing by single-copy marker genes, plasmid abundance ranged from 0.02 to 1.65 copies per cell, with significantly higher abundance in the PA fraction (DYB: *P* = 0.015; PRE: *P* < 0.001; Fig. 2c). The PA fraction contained 0.097 copies per cell of mobilizable/conjugative plasmids (19.0% of total plasmid abundance), whereas the FL fraction contained only 0.0185 copies per cell (13.2%; Fig. 2d and e). Both absolute and relative abundances of mobilizable/conjugative plasmids were significantly higher in the PA fraction than in the FL fraction (*P* < 0.015; Fig. 2d and e). Besides, significant differences between DYB and PRE were also observed in plasmid abundance, the number of observed PTU, and mobilizable/conjugative plasmid abundance (Fig. 2b through e).

Host prediction indicated that all PTUs originated from bacteria, dominated by Pseudomonadota (87.2%), followed by Bacillota (5.5%) and Actinomycetota (3.3%; Fig. 2a). Fewer than 15 PTUs were assigned to Thermodesulfobacteriota, Bacteroidota, Spirochaetota, Deinococcota*,* and Cyanobacteriota. Pseudomonadota could be further separated into Alphaproteobacteria (44.2%), Gammaproteobacteria (34.3%), and Betaproteobacteria (6.7%). Across samples, both PA and FL plasmidomes were mainly associated with Gammaproteobacteria, Betaproteobacteria, and Alphaproteobacteria (Fig. 2f). The PA fraction harbored a higher relative abundance of plasmids from Betaproteobacteria (*P* < 0.001), while FL fractions had a higher relative abundance of plasmids from Alphaproteobacteria (*P* < 0.001; Fig. 2f), consistent with bacterial community composition (Fig. S3d). ANOSIM further confirmed significant differences between PA and FL plasmidome structures in both PRE and DYB (*P* < 0.001; Fig. S5).

**Fig 3:**
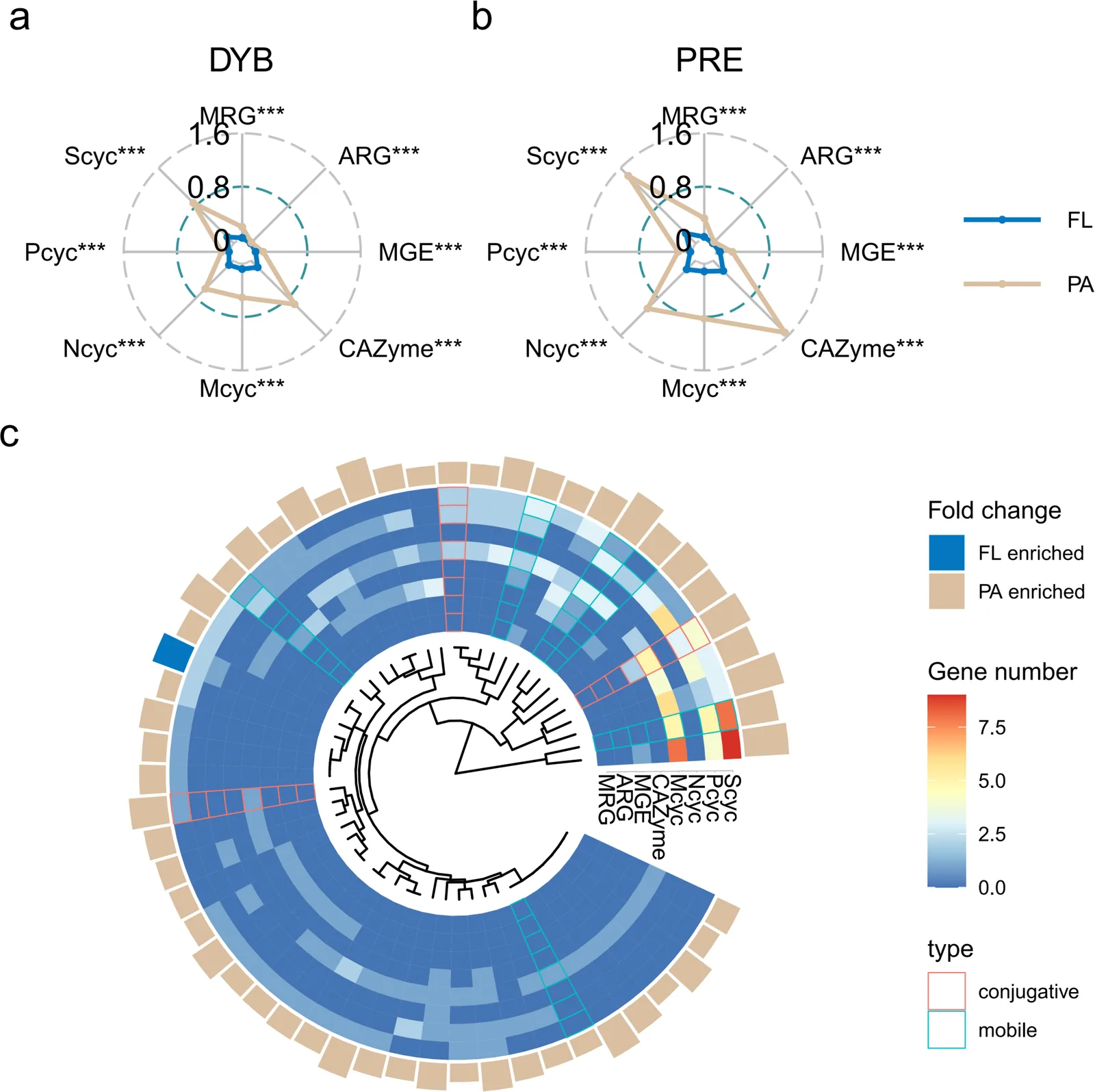
Plasmid-carried functional gene abundance profiles and PTU enrichment across particle-size fractions in DYB and PRE. Radar charts showing the abundance (copies per cell) of eight categories of plasmid-carried functional genes in particle-attached (PA) versus free-living (FL) fractions across two regions, (**a**) DYB and (**b**) PRE. Asterisks following gene categories indicate significant differences between PA and FL fractions, Wilcoxon rank-sum test: ***P < 0.001. (**c**) The tree was constructed based on the similarity of functional genes carried by each PTU. Branch colors indicate the enrichment of PTUs between particle-attached (PA) and free-living (FL) fractions: red denotes significant enrichment in the PA fraction, and blue denotes significant enrichment in the FL fraction (differential analysis by DESeq2, fold change > 20). Only PTUs harboring at least one of the key functional genes are shown. CAZyme, carbohydrate-active enzymes; MCcyc, methane cycling genes; MGE, mobile genetic elements (mainly transposases, integrases, and insertion sequences); MRG, metal resistance genes; Ncyc, nitrogen cycling genes; Pcyc, phosphorus cycling genes; Scyc, sulfur cycling genes.

Considering functional traits of plasmids, the PA plasmidome exhibited larger abundance-weighted genome sizes and higher gene counts than the FL plasmidome (Fig. S6), indicating a greater genetic contribution to host communities. In total, 9,631 non-redundant putative genes were identified across PTUs, including genes from carbohydrate-active enzyme genes (CAZyme), methane cycling genes (MCcyc), nitrogen cycling genes (Ncyc), sulfur cycling genes (Scyc), phosphorus cycling genes (Pcyc), ARGs, MRGs, and MGEs (Fig. S7). In total, 574 PTUs, more than half of the detected PTUs, carried at least one of these functional genes. Similar to higher genome size and more genes, the PA plasmidome contained significantly higher abundances of genes from multiple functional categories than the FL plasmidome (Fig. 3a and b). Overall, PA plasmidomes were enriched in functional genes.

Differential abundance analysis (DESeq2) identified 121 significantly enriched PTUs (fold change >20, FDR adjusted *P* < 0.05), including 111 PTUs significantly enriched in the PA fraction and 10 PTUs enriched in the FL fraction. 13 out of 111 PTUs enriched in the PA fraction were mobilizable or conjugative, while all PTUs enriched in FL fractions were non-mobilizable. Among PTUs showing significant differences in the two fractions, 55 PTUs carried one or more AMGs, resistance genes, or MGEs, and almost all of these plasmids were enriched in the PA fraction (Fig. 3c).

### Mechanisms driving plasmid diversity

PREs were relatively affected by stronger anthropogenic activities than DYB (Fig. S2), and also exhibited higher abundance and diversity of plasmids (Fig. 1b through e), suggesting that anthropogenic activities may increase plasmid diversity. Subsequently, we used PC1 to represent the changes in environmental indicators between different samples, as PC1 explained the 62.8% variations of environmental variables (Fig. 1a). PC1 was positively correlated with the number of observed PTU (*P* = 0.028; Fig. S8a), indicating that environmental variation contributed to shaping plasmid diversity.

Considering host diversity, the number of observed PTUs was not significantly correlated with the number of observed bacterial ASVs (*P* = 0.553; Fig. S8b). We found a significantly positive correlation between the number of observed genera and families with the observed number of PTUs (genus: *P* = 0.024; family: *P* = 0.023; Fig. S8c and d) and the number of observed families showing better fitness than the number of observed genera. This suggested that PTUs defined at 95% identity and 80% coverage may correspond broadly to genus- or family-level host lineages, instead of ASVs level.

For biofilm-formation genes (BFGs), we identified 202 different genes involved in the biofilm formation pathway, from KEGG level 3, that is, Biofilm formation*—Vibrio cholerae*, Biofilm formation*—Escherichia coli*, and Biofilm formation*—Pseudomonas aeruginosa* (Table S1). After normalizing by single-copy marker genes, the PA fraction owned 19.99 copies BFGs per cell, exhibiting significantly more abundant BFGs per cell than the FL fraction (13.85 copies BFGs per cell; *P* < 0.001). BFGs abundance was strongly correlated with the number of observed PTUs (*P* < 0.001; Fig. S8b), indicating a potential role in structuring plasmid diversity.

**Fig 4:**
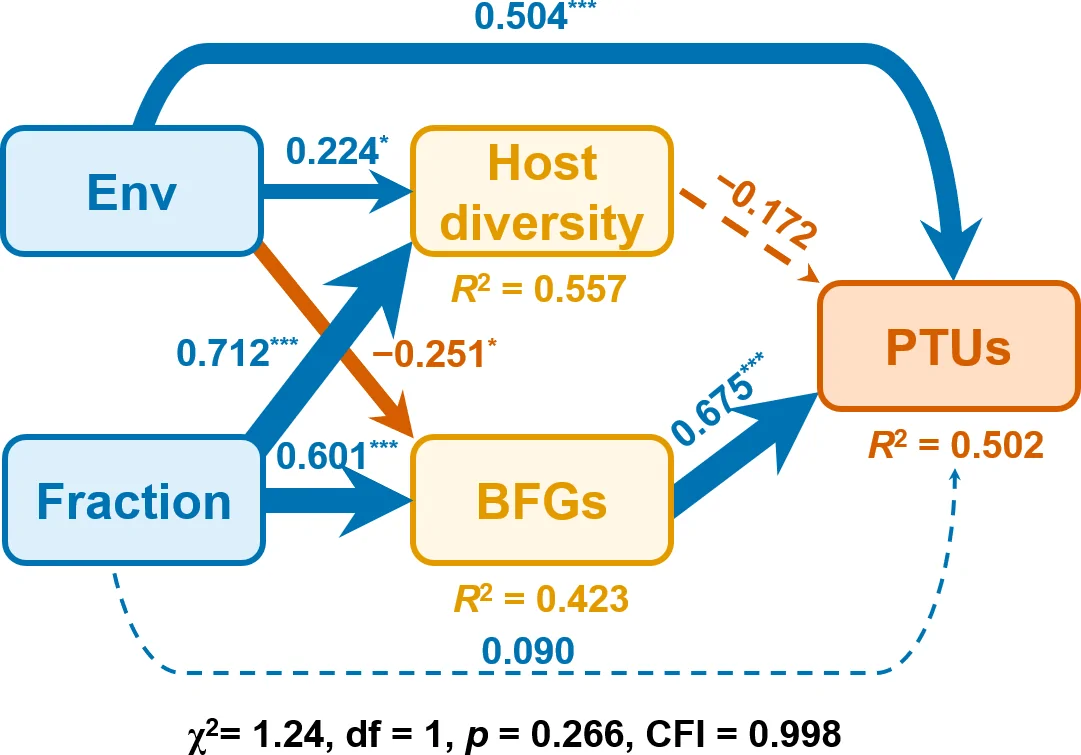
Structural equation model illustrating how environmental gradient and particle-size fractions influence plasmid diversity via host diversity and biofilm-forming genes. This model elucidates the potential paths through which the environmental gradient (Env, represented by PC1) and different size fractions (PA as 1 and FL as 0) drive plasmid richness (represented by the number of observed PTUs), by influencing host bacterial diversity (at the family level) and the abundance of biofilm-forming genes (BFGs). The model was constructed and tested using the “lavaan” package in R. Arrows in the diagram represent hypothesized causal relationships. Blue lines represent positive paths, and red lines indicate negative paths; solid lines represent significant paths (*P* < 0.05), and dashed lines indicate paths that were not statistically significant (*P* > 0.05). The numbers above the arrows are standardized path coefficients, with asterisks denoting the significance level (***P < 0.001, and *P < 0.05). The thickness of lines also represents standardized path coefficients. The overall model fit indices are shown below the figure.

To disentangle these drivers, we constructed a SEM based on hypothesized processes (Fig. S9). We proposed that size fraction and environmental gradients (PC1) first shaped host taxonomic diversity (family level) and BFGs, and further affected plasmid diversity (the number of observed PTUs) through direct and indirect paths. The model showed good fitness (*χ*^2^ = 1.23, df = 1, *P* = 0.266, CFI = 0.998; Figure 4). Overall, size fractions (total effects = 0.372) and environmental gradients (total effects = 0.297) had comparable effects on plasmid diversity. Enrichment of plasmids in the PA fraction was primarily mediated by BFG abundance, accounting for 65.3% of the total effect of size fraction. In contrast, environmental gradients acted mainly through direct effects, accounting for 70.9% of the total effect of PC1.

**Fig 5:**
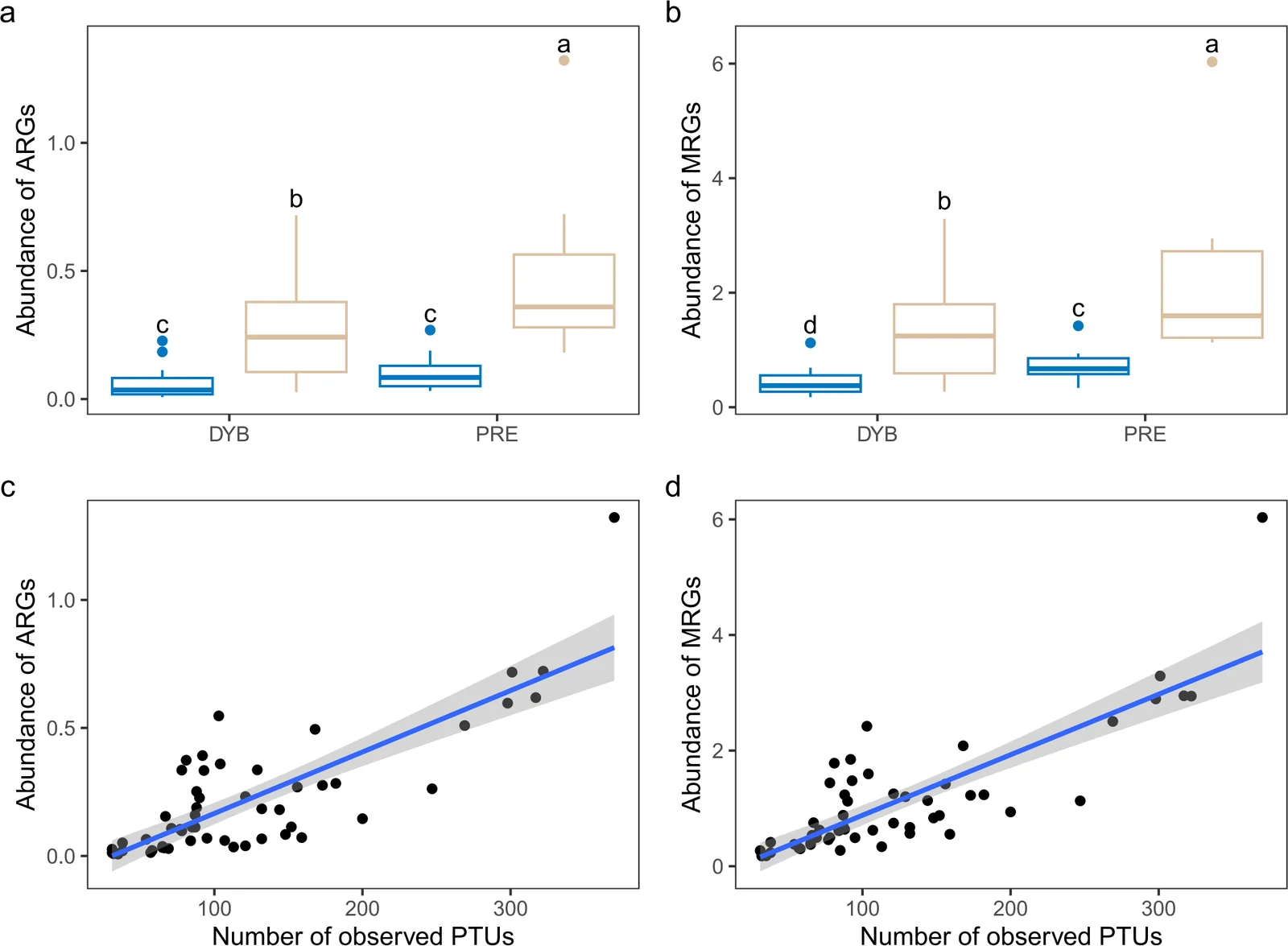
The distribution of the resistome and their relationships with plasmid diversity. Box plots showing the average gene abundance normalized per cell of (**a**) antibiotic resistance genes (ARGs) and (**b**) metal resistance genes (MRGs) across different regions and fractions. Pairwise comparisons between groups were performed using the Wilcoxon rank-sum test, with *P* values adjusted by the false discovery rate method. Different letters indicate statistically significant differences between groups. Linear regression showing plasmid diversity, represented by the number of observed PTUs, and (**c**) ARGs and (**d**) MRGs. The shaded area represents the 95% confidence interval of the regression line.

### Linking plasmids to the resistome

A total of 12 ARG types and 15 MRG types were detected in PRE and DYB (Fig. S10). Multidrug resistance and bacitracin resistance were the dominant ARG types, while mercury and arsenic resistance were the dominant MRG types (Fig. S10). In both PRE and DYB, the PA fraction showed significantly higher abundance of ARG and MRG than FL fractions (Fig. 5a and b). We identified 4 PTUs and 71 PTUs carried at least one ARG and MRG, respectively, and these plasmid-derived genes accounted for 8% and 23.9% of total ARGs and MRGs abundance, respectively. Despite the low contribution of plasmids to ARGs and MRGs, the number of observed PTUs and plasmid abundance showed a strong correlation with ARGs and MRGs abundance (Fig. 5c and d). Although plasmid distribution between different size fractions was strongly influenced by BFGs, the number of observed PTUs (ARG: Pearson’s *r* = 0.792; MRG: Pearson’s *r* = 0.777) showed correlations with ARGs and MRGs comparable to those of BFGs (ARG: Pearson’s *r* = 0.834; MRG: Pearson’s *r* = 0.830).

## DISCUSSION

### Potential mechanisms of enrichment of plasmids in PA environments

Our comparative analysis revealed that PA bacteria harbored significantly greater plasmid diversity and abundance than FL bacteria. SEM further demonstrated that BFGs were the key factors in shaping the pattern of plasmid diversity in PA and FL, beyond host diversity. This finding pointed to a fundamental ecological explanation beyond host composition: spatial architecture. PA communities are characterized by spatial aggregation and structured colonization of microorganisms on particulate surfaces, forming a low-entropy microenvironment with high local cell density; in contrast, FL microorganisms are relatively evenly distributed throughout the water column, exhibiting high entropy characteristics in space (25, 31). This unique spatial structure could provide the physical and ecological foundation for plasmid dispersal. Previous theoretical models and experimental work have shown that conjugation efficiency is proportional to the square of local plasmid donor–recipient cell density (25). Consequently, the high-density microenvironment of the PA fraction likely promotes frequent plasmid exchange, explaining the elevated plasmid diversity and abundance observed in PA communities.

Spatial aggregation does not occur spontaneously, but is mediated by the BFGs, leading to higher cell density. We observed a significant positive correlation between BFG abundance and plasmid diversity, consistent with the selective pressure of PA environments for biofilm-like traits (31). Indeed, PA habitats favor genes involved in polysaccharide biosynthesis, secretion systems (including the general secretory pathway and type VI secretion system), MSHA pilin, two-component regulatory systems, quorum-sensing regulators, and genes encoding extracellular polymeric substances (EPS; Table S1) (31). BFGs may facilitate the transition to a PA lifestyle through three primary mechanisms: (i) Adhesion and colonization basis: BFGs encode EPS synthesis machinery and specialized pili, providing a physical scaffold for initial attachment and stable aggregation on particles. (ii) Environmental sensing and response: two-component regulatory systems enable cells to detect chemical gradients and physical conditions at particle interfaces and initiate attachment-related gene expression. (iii) Coordination of community behavior: quorum-sensing allows high-density populations to coordinate EPS production, metabolic specialization, and resistance expression, thereby stabilizing aggregated structures. Overall, BFGs largely cover genes related to the formation of high-density environments, later microbial adaptation, and interspecies interactions, thereby promoting the persistence of high-density environments.

Our SEM specifically focused on how the abundance of BFGs and host diversity explained the variance of plasmid diversity. However, some mechanisms that are difficult to quantify were not included in this model. For example, previous studies have highlighted heterogeneity of suspended particles and nutrient enrichment in particles as important mechanisms for promoting bacterial diversity in the PA fraction (6). While these factors may indirectly influence the plasmidome by supporting a diverse host background, our SEM indicates that this indirect path was relatively weak. Furthermore, the lack of a significant direct path from size fraction differences to plasmid diversity suggested that their isolated impact is secondary. Ultimately, the SEM result provided robust evidence that BFG-mediated spatial aggregation is the dominant driving force shaping higher plasmid diversity in the PA fraction.

### Helping to understand genomic architecture and niche partitioning between PA and FL

PA and FL bacteria exhibit a fundamental divergence in evolutionary strategy at the chromosomal view that underlies their functional differentiation. Typical FL bacteria, such as the SAR11 clade, follow a “streamlined genome” strategy. They minimize maintenance costs and adapt to homogeneous, nutrient-poor aquatic environments by reducing non-essential genes related to complex regulation, motility, and signal transduction (32). In contrast, PA bacteria generally possess larger genomes and more expansive gene pools. These genomes are enriched with genes for specialized substrate utilization (e.g., CAZyme), environmental sensing (e.g., two-component systems), and intercellular interactions (4). These chromosomal traits overlap substantially with the BFGs identified, supporting microbial colonization and competition at particle interfaces.

Our findings further extended this functional differentiation pattern to the plasmid dimension. The abundant plasmids in the PA community conferred a few functions for adapting to particulate habitats, which could help them enhance fitness for the host. First, plasmid-borne CAZymes address the two primary challenges of the PA lifestyle: acquiring metabolic substrates and maintaining stable physical attachment. CAZymes enable the hydrolysis of complex polysaccharides within particulate organic matter, providing a direct carbon source. Simultaneously, some endogenously synthesized polysaccharides are precursors for biofilm matrix construction and serve as precursors for biofilm matrix formation (33). Second, plasmid-borne element cycling genes help adapt to the microenvironment in the particle, particularly oxygen and nutrients. The enrichment of phosphorus-cycling genes on PA plasmids may correspond to the dominance of particulate phosphorus in coastal waters (34), thereby providing PA bacteria a competitive advantage. Furthermore, the presence of redox genes involved in the methane, nitrogen, and sulfur cycles suggests that plasmids enable microorganisms to rapidly occupy specialized niches, such as denitrification or sulfate reduction, within the particle core’s anaerobic microenvironments (6).

Beyond their direct role in harboring functional genes, plasmids, as a prominent class of MGEs, interact with other non-plasmid MGEs to dynamically host gene acquisition. Our study highlights the PA environment as a specialized incubator for these interactions, driven by two primary mechanisms. First, the spatial structure of the PA fraction enhances conjugation by increasing cell–cell contact. We observed significantly higher proportions of mobilizable/conjugative plasmids, as well as higher abundances of integrons and transposons, in the PA fraction than in the FL fraction (Fig. 1d and e; Fig. S9 and S10). This dense microenvironment maintains an active plasmid pool and facilitates rapid dissemination of advantageous traits. Second, MGEs are consistently enriched in PA communities (4, 19, 22), and their abundance is positively related to genome size (35, 36). This enrichment provides a genetic basis for the larger genomes of PA bacteria and supports a more flexible functional repertoire than that of streamlined FL bacteria (19).

Furthermore, the EPS matrix, synthesized by the BFGs, provides crucial biophysical infrastructure for HGT pathways beyond conjugation (37). (i) Transformation: the EPS matrix traps and protects extracellular DNA from enzymatic degradation, creating a reservoir for uptake by competent cells. (ii) Transduction: the biofilm microenvironment provides shelter for bacteriophages, increasing the likelihood of persistent infection and the transfer of bacterial genes. Extracellular DNA released by phage-mediated lysis further promotes the transformation process.

### Coupling mechanisms of human activities, plasmid diversity and resistome

Our research indicated that anthropogenic activities influence not only plasmid diversity but also its association with ARGs and MRGs. SEM confirmed that PC1, representing environmental gradients and human impact, exerted a significant direct effect on plasmid diversity. Spatial patterns support this interpretation: the PRE, strongly affected by urbanization and industrial and agricultural discharges, exhibited higher nutrient concentrations (DON and DIP), petroleum hydrocarbons, and chemical oxygen demand than DYB. These findings are consistent with previous reports of elevated PAHs (38) and heavy metals (28) in the PRE compared with the DYB, largely due to greater riverine inputs in PRE. Consequently, salinity showed a negative relationship with anthropogenic disturbance, as high-salinity marine water dilutes pollutant loads.

Although a strong correlation between plasmid diversity and the abundance of ARGs and MRGs was observed (Fig. 5c and d), relatively few resistance genes were directly recovered on assembled plasmids. In fact, the strong correlation between plasmid diversity and the abundance of ARGs and MRGs has been observed in other metagenomic studies (24, 39). It suggests that the link between plasmids and the resistome is far from accidental, likely driven by anthropogenic activities (24, 39, 40). Two explanations could be proposed for this observation: (i) Incomplete recovery of plasmids containing ARGs and MRGs. While plasmids are critical vectors for the resistome, their recovery from complex environmental metagenomes remains a significant technical challenge (41). Low coverage of rare plasmids, assembly fragmentation, and limited marker genes may lead to underestimation of resistance-carrying plasmids. Increased sequencing depth and plasmid-targeted extraction methods may reveal a more complete plasmid-borne resistome (19, 40). (ii) Coupled selection of plasmids and the resistome. Recent evidence suggests that environmental concentrations of antibiotics (42), heavy metals (43), and even pollutants like pesticides (44) significantly increased cell membrane permeability and conjugation efficiency. Under such scenarios, pollutants do not only select individuals carrying resistance genes, but also increase the plasmid transfer efficiency across the entire community. This spread process could be further amplified at the particulate matter interface. These organic pollutants and metals preferentially adsorb onto particulate surfaces, creating higher local concentrations than surrounding water (45). Simultaneously, the formation of biofilms also helps microorganisms resist external stresses. This chemical enrichment was coupled with the high BFGs abundance in PA communities, resulting in a strong correlation between plasmid diversity and the abundance of MRGs and ARGs. As a result, we found a higher correlation between the number of PTUs and the abundance of ARGs and MRGs in the PA fraction than in the FL fraction. Ultimately, plasmids and resistomes show a concurrent increase along the human activity gradient.

### Conclusion

This study provided a comprehensive plasmid-centric analysis of particle-attached (PA) and free-living (FL) bacterial communities in PRE and DYB regions. We demonstrated that PA communities harbor significantly higher plasmid diversity and abundance than FL communities, and this phenomenon was primarily driven by their spatial structure rather than host diversity, as supported by SEM. In addition to directly providing auxiliary metabolic genes, plasmids may promote functional differentiation between PA and FL bacterial communities by facilitating horizontal gene transfer, particularly leading to a larger genome size in PA bacteria. Our findings also highlighted the profound impact of human activities on coastal plasmidomes; heavily polluted areas, such as PRE, are key hotspots for plasmids and resistance genes. The strong correlation between plasmid diversity and the abundance of antibiotic and metal resistance genes suggests that plasmids may promote the spread of antibiotic resistance genes through horizontal gene transfer. These results emphasize that particulate-attached microorganisms in polluted areas may serve as hotspots for the dissemination of resistance genes. Overall, this plasmid-centered metagenomics study is significant for understanding the functional differentiation and environmental adaptation of microorganisms between PA and FL communities, as well as revealing potential dispersal mechanisms of the resistome in aquatic bodies.

## MATERIALS AND METHODS

### Field sampling, measurement of environmental variables, and collecting historical data

Two cruises were conducted in the PRE and DYB, respectively, in early December 2024 (Fig. S1), as the dry season was normally accompanied by higher pollution levels. Surface seawater (1 m depth) was collected from 33 sites using Niskin bottles. The samples were transported to the laboratory in an insulated container with ice (<6 h) until laboratory processing. For microbial community analysis, 5 L of pre-filtered seawater was sequentially filtered through 3.0 µm (PA) and 0.2 µm (FL) polycarbonate membranes (Millipore, USA) under low vacuum. Filters were immediately flash-frozen in liquid nitrogen and stored at −80°C until DNA extraction.

For nutrient analysis, subsamples were filtered through GF/F (Waterman, USA) cellulose acetate filters, and the filtrates were stored at −20°C. *In situ* water temperature, salinity, pH, and dissolved oxygen were measured using a YSI ProDSS sensor (YSI, USA). Concentrations of dissolved total nitrogen, DIP, DOC, dissolved total phosphorus, and dissolved total nitrogen were analyzed with a continuous flow analyzer (Seal AA3, Germany). DON (DOP) were calculated as the difference between dissolved total nitrogen

To characterize long-term anthropogenic pressure in the PRE and DYB, historical monitoring data (2017–2025) were retrieved from the Chinese National Marine Environmental Monitoring Center database (http://ep.nmemc.org.cn:8888/Water/). This data set includes two to three surveys annually and reports pH, dissolved oxygen, chemical oxygen demand, DIN, DIP, and petroleum hydrocarbons following the Chinese Seawater Quality Standard (GB3097-1997), with water quality classified into Grades I–IV and inferior to Grade IV. Monitoring stations within the PRE and DYB regions were selected, and COD, petroleum hydrocarbons, and water quality grades were used as indicators of anthropogenic impact.

### DNA extraction and sequencing

DNA was extracted from filters using the FastDNA Spin Kit (MP Biomedicals, USA) following the manufacturer’s protocol. DNA concentration and purity were assessed using a NanoDrop spectrophotometer (Thermo Fisher Scientific, USA). Several PA samples yielded insufficient DNA for metagenomic sequencing; therefore, both PA and corresponding FL fractions from these sites were excluded from subsequent analyses. High-quality DNA was used for library preparation.

The V4 region of the 16S rRNA gene was amplified with primers 515F (5′-GTGCCAGCMGCCGCGGTAA-3′) and 806R (5′-GGACTACHVGGGTWTCTAAT-3′). PCR conditions were: 95°C for 2 min; 25 cycles of 95°C for 30 s, 55°C for 30 s, 72°C for 30 s; and a final extension at 72°C for 5 min. Each reaction (20 µL) contained 4 µL 5 × FastPfu Buffer, 2 µL 2.5 mM dNTPs, 0.8 µL of each primer (5 µM), 0.4 µL FastPfu Polymerase, and ~10 ng template DNA. Amplicons were gel-purified with the AxyPrep DNA Gel Extraction Kit (Axygen Biosciences, USA).

Shotgun metagenomic libraries were constructed using the TruSeq DNA Library Preparation Kit (Illumina, USA) and quantified with the Qubit High Sensitivity dsDNA Assay Kit (Thermo Fisher Scientific, USA). Libraries were sequenced on an Illumina HiSeq 2000 platform (2 × 150 bp paired-end) at Shanghai Biozeron Biotechnology Co., Ltd. Negative controls (extraction blanks, *n* = 6) were included during library preparation but failed quality control and were not sequenced.

All raw sequencing data have been deposited in the China National Center for Bioinformation under accession number PRJCA045552.

### Bioinformatic analysis

Raw 16S rRNA gene reads were quality filtered using Trimmomatic (46), and primer sequences were removed with Cutadapt (47). Denoising and ASVs inference were performed using DADA2 (48), with chimeras removed during processing. Taxonomic assignment was conducted using the SINTAX classifier against the RDP v19 database (49) with a confidence threshold of 0.8. Only bacterial ASVs were retained; archaeal and unclassified ASVs were excluded. All samples were rarefied to 45,000 reads before downstream analyses.

Shotgun metagenomic reads were processed in several steps. First, KneadData (https://huttenhower.sph.harvard.edu/kneaddata/) was used to remove low-quality bases, adapter sequences, and potential host sequences against the human genome database. This quality filtering process afforded 28–41 million paired-end clean reads. Cleaned reads were assembled into contigs using MEGAHIT with “--min-contig-len 500” parameters (50). Circular sequences >1 kb were screened with geNomad, and non-circular contigs were also evaluated with geNomad to recover additional plasmid-derived sequences (41), and non-circular contigs were also evaluated with geNomad to recover additional plasmid-derived sequences (41). Circular plasmids were reserved with sequences >1 kb in length, FDR < 0.05, and containing direct terminal repeats (DTRs). Plasmid fragments were reserved as sequences >1 kb, with FDR < 0.05, lacking DTRs but containing at least one plasmid hallmark gene. Circular plasmids and plasmid fragments were clustered into putative plasmid taxonomic units (PTUs) using CheckV scripts anicalc.py and aniclust.py (51), with thresholds --min_ani 95 --min_tcov 80 --min_qcov 0 (52). Host information for PTUs was inferred using HOTSPOT, a deep learning framework that predicts plasmid-host associations from sequence features (53); this tool provides a similar annotation system as RDP v19. The determination of complete conjugative systems (relaxases, T4SS ATPases, T4CPs, and other T4SS components) was based on the presence of components using the CONJscan model (54, 55). Mobilizable plasmids contained at least one relaxase, and conjugative plasmids contained all of the relaxase, the T4SS ATPase, and the T4CP.

All samples were first resampled to 28 million paired-end reads to exclude the influence of sequence reads on diversity assessment. The PTU coverage across samples was quantified using SMAtools (56), and a PTU was considered present if ≥80% of its bases were covered by at least once in a sample, with their relative abundances expressed in transcripts per million (TPM) (39). Open reading frames (ORFs) in representative PTU sequences were predicted using Prodigal (57) and clustered with CD-HIT (95% identity and 90% coverage) to generate a nonredundant plasmid gene catalog (58), which was defined as plasmid-derived genes. Gene abundance was calculated as the mean coverage of plasmid PTUs to avoid overestimation due to highly conserved homologs.

Functional annotation focused on three categories of ecological relevance: (i) AMGs included CAZyme, MCcyc, Ncyc, Scyc, and Pcyc. Those genes were annotated against the CAZyme families (59), MCcycDB (60), NcycDB (61), ScycDB (62), and PcycDB (63) using DIAMOND (64). (ii) Resistance genes included ARGs and MRGs; these genes were annotated against the SARG v2.3 (65) and the BacMet (66) database. (iii) MGEs, including insertion sequences, transposons, and integrons. These genes were annotated against the MGEs database (67). All annotations were performed based on the BLASTP algorithm, applying query cover and subject cover larger than 40%, identity larger than 70%, and an *e*-value cutoff of 10^−5^.

ORFs predicted from total metagenomic contigs were additionally annotated against the KEGG, SARG, and BacMet databases. Gene abundances were normalized to copies per cell using 35 universal single-copy marker genes (68).


(1) $$copies\;per\;cell=\frac{\sum Reads*L_{r}/L_{g}}{N}$$

where *N* is cell number estimated from the average coverage of 35 universal single-copy marker genes, *L*_g_ is average ORF length, *L*_r_ is read length, and *D* is sequencing depth.

### Statistical analysis

Environmental variables were log-transformed prior to analysis. Principal component analysis (PCA) was applied to visualize environmental gradients, and environmental variables with a contribution value of less than 0.1 to both PC1 and PC2 were excluded; multiple response permutation procedures were used to test differences between PRE and DYB.

Nonmetric multidimensional scaling analysis for plasmid and host community based on Bray-Curtis’s dissimilarity was performed using “vegan” package. Analysis of similarities (ANOSIM) was used to test the significance of the differences in community composition, employing 9,999 permutations to evaluate the *P* values using “vegan” package. To identify PTUs that differed significantly in abundance between PA and FL fractions, we applied “DESeq2” package (69) using reads count data, with fraction type specified as the main factor in the model. All raw count data plus 1 as “DESeq2” package can’t handle zero count data. PTUs with a false discovery rate (FDR) adjusted *P* < 0.05 and a fold change > 20 were considered significantly enriched.

To understand how environmental gradients and different size fractions influence plasmid abundance through host diversity and BFGs abundance, we constructed a structural equation model based on the hypothetical pathway (Fig. S9). The model was constructed using the “lavaan” package and statistically tested. The chi-square test was used to test model significance, and the CFI was used to evaluate goodness of fit. The standard deviations of each coefficient were calculated based on 1,000 bootstrap samplings. The number of observed families was used to represent host diversity as they showed higher correlation with the number of observed PTUs than the number of observed families genera.

All statistical analyses were conducted in R-4.4.3 (https://www.r-project.org), and the figures were visualized using the “ggplot2” packages unless otherwise stated.

## Data Availability

All raw sequencing data have been deposited in the China National Center for Bioinformation under accession number PRJCA045552.

## SUPPLEMENTAL MATERIAL

Supplemental figures spectrum.00460-26-s0001.docx Fig. S1 to S10.

Table S1 spectrum.00460-26-s0002.xlsx The number of biofilm-forming genes identified in this study and their function information.

ASM does not own the copyrights to Supplemental Material that may be linked to, or accessed through, an article. The authors have granted ASM a non-exclusive, world-wide license to publish the Supplemental Material files. Please contact the corresponding author directly for reuse.
