# Supplementary material for: Biofilm-forming traits enrich the plasmid diversity and functional potential in particle-attached bacteria in coastal ecosystems
Source: Microbiol Spectr. 2026 Jun 15;14(7):e00460-26. doi: 10.1128/spectrum.00460-26 (PMC13340213; doi:10.1128/spectrum.00460-26)
Supplement: Supplemental figures — Fig. S1 to S10. [file spectrum.00460-26-s0001.docx]

**Biofilm-forming traits enriched the plasmid diversity and functional potential in particle-attached bacteria in coastal ecosystems**

Zhendu Mao ^a^, Mengying Jiang ^a,c^, Zifan Zhao ^a^, Shumin Xu ^a^, Heng Wang ^a,d^, Kelin Chen ^a,e^, Jianglang Duan ^a,f^, Zhuo Chen ^a,f^, Dan He ^a^, Peng Xing^b^, Qinglong L. Wu ^b,a,g,h,#^

^a^ Center for Evolution and Conservation Biology, Southern Marine Sciences and Engineering Guangdong Laboratory (Guangzhou), Guangzhou, China

^b^ Key Laboratory of Lake and Watershed Science for Water Security, Nanjing Institute of Geography and Limnology, Chinese Academy of Sciences, Nanjing, China

^c^ Department of Ocean Science and Engineering, Southern University of Science and Technology, Shenzhen, China

^d^ School of Life Sciences, Institute of Life Science and Green Development, Hebei University, Baoding, China

^e^ Department of Ecology and Institute of Hydrobiology, Jinan University, Guangzhou, China

^f^ School of Environmental Science & Engineering, Guangzhou University, Guangzhou, China

^g^ Sino-Danish Center for Science and Education, University of Chinese Academy of Sciences, Beijing, China

^h^ Lake Fuxian Ecological Research Station, Chinese Academy of Sciences, Chengjiang, China

# Author for correspondence. Qinglong L. Wu, E-mail: qlwu@niglas.ac.cn, Phone: +86-25-86882107

**Running title:** differences between PA and FL plasmidomes


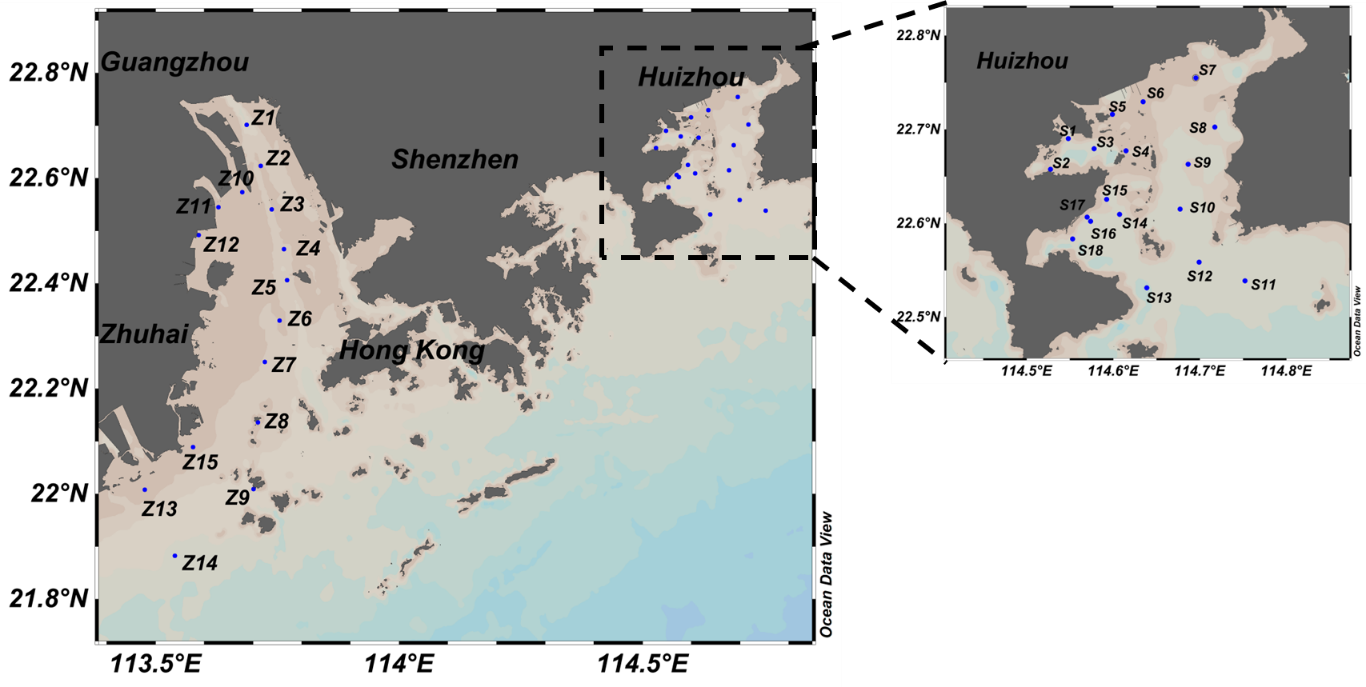


**Fig. S1.** Sampling locations in the Pearl River Estuary (PRE) and Daya Bay (DYB). PRE includes 15 sites (Z1-Z15), and DYB includes 18 sites (S1-S18). DNA samples >3 μm from S1, S3, Z2, Z9, Z12, and Z14 did not meet the quality requirements for metagenome sequencing and were excluded from further analysis.


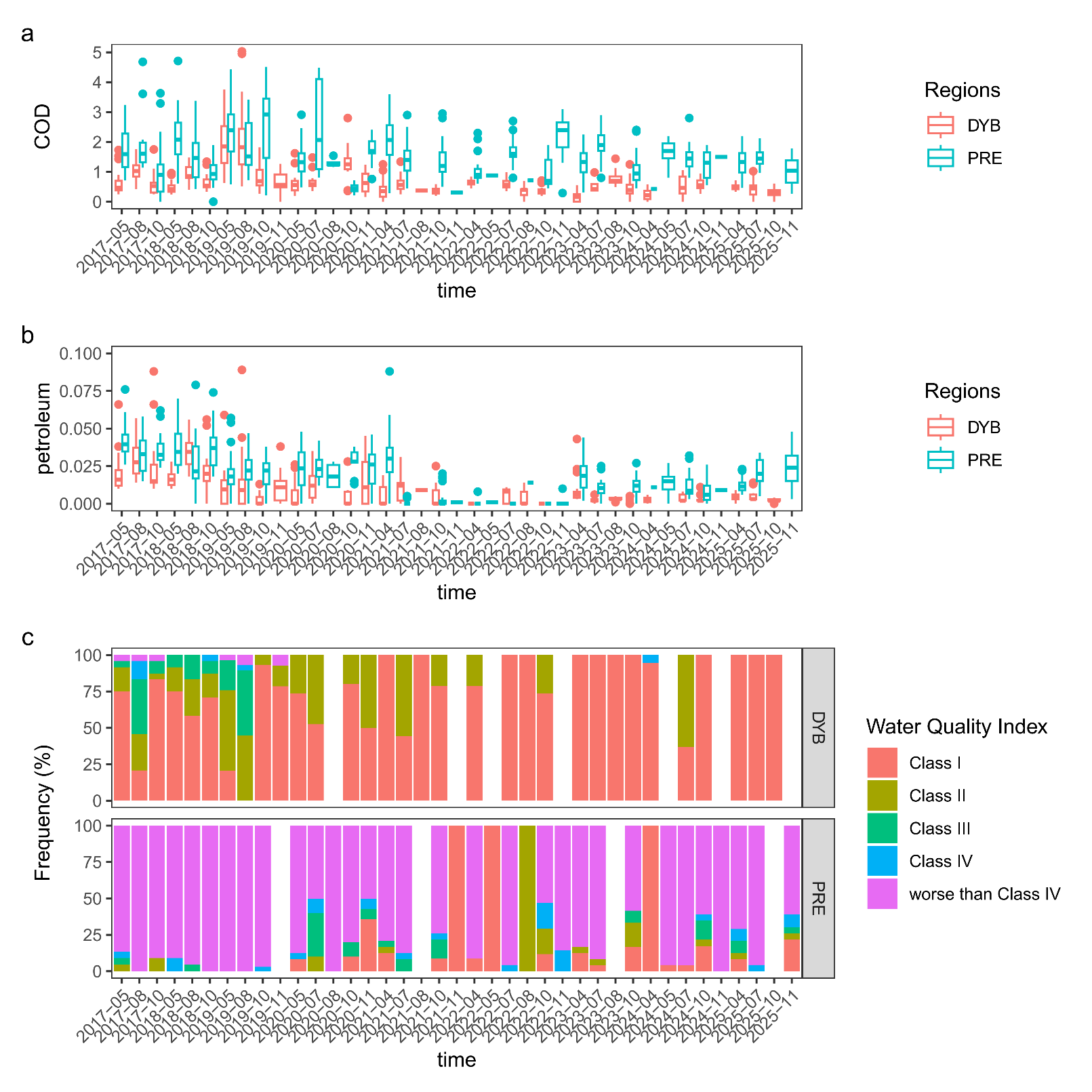


**Fig. S2**. Historical trends of water quality parameters in DYB and the PRE from 2017 to 2025. Historical monitoring data was collected from 2017 to 2025 (with 2-3 surveys per year), comparing the long-term water quality variations between DYB and PRE. (a) Inter-annual variation in chemical oxygen demand concentration (COD); (b) Inter-annual variation in petroleum hydrocarbon content; (c) Distributions of water quality grades at sampling sites, assessed according to the Chinese Water Quality Standard. Class I to Class IV indicate a sequential decline in water quality from good to poor, with “worse than Class IV” representing severely polluted conditions.


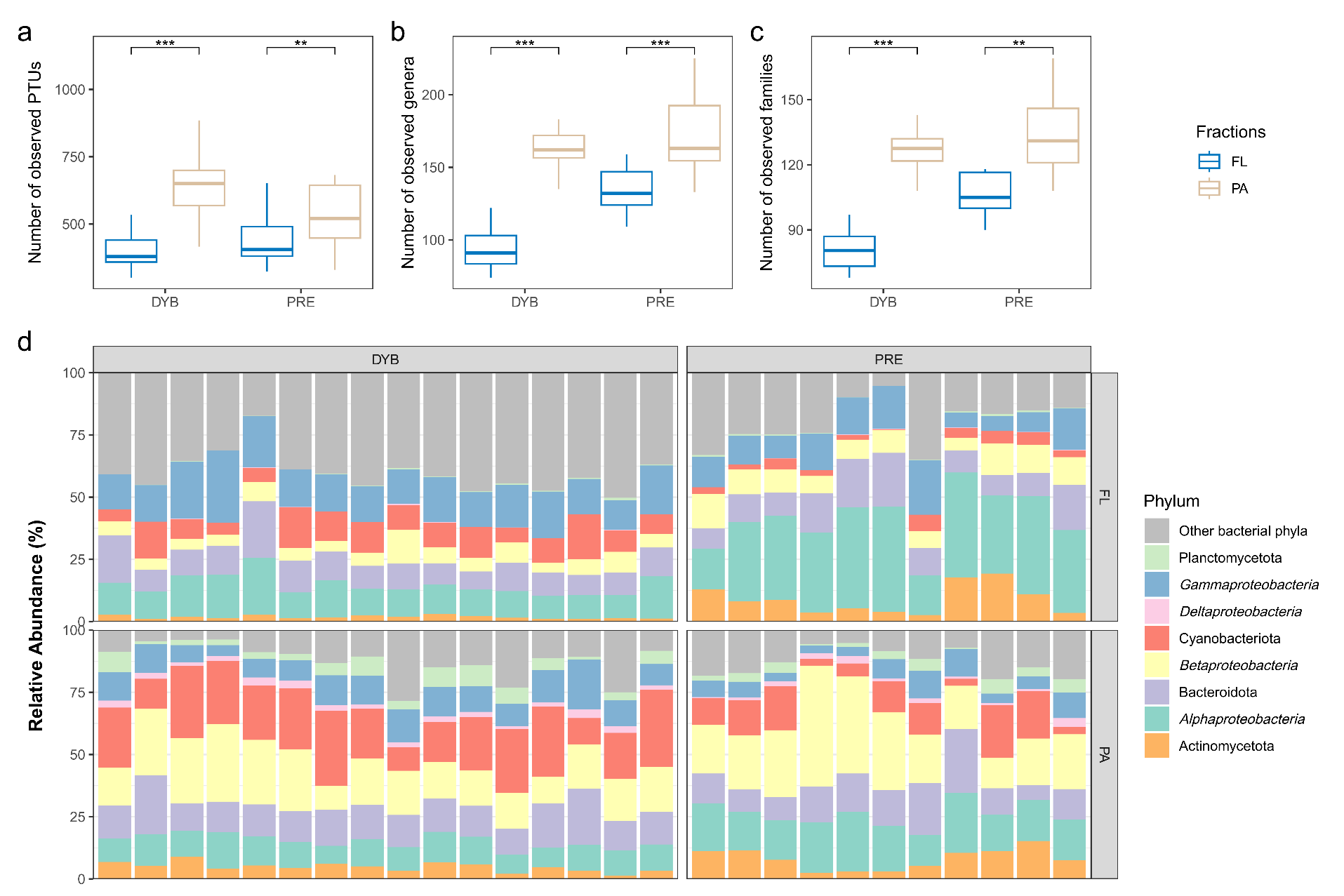


**Fig. S3.** Differences in microbial community diversity and composition across particle-size fractions from DYB and PRE. Box plots showing comparisons of microbial diversity, including (a) number of observed amplicon sequence variants (ASVs), (b) number of observed genera (c) Number of observed families between regions and fractions. Comparisons between groups were performed using t-tests, and asterisks above indicate the significance level: ****p* < 0.001, ***p* < 0.01, **p* < 0.05). (d) Relative abundance of bacterial communities at the phylum level for each sample. Only phyla with average abundance > 1% were shown here. The phylum Pseudomonadota was separated into *Alphaproteobacteria*, *Betaproteobacteria*, *Gammaproteobacteria*, and *Deltaproteobacteria*.


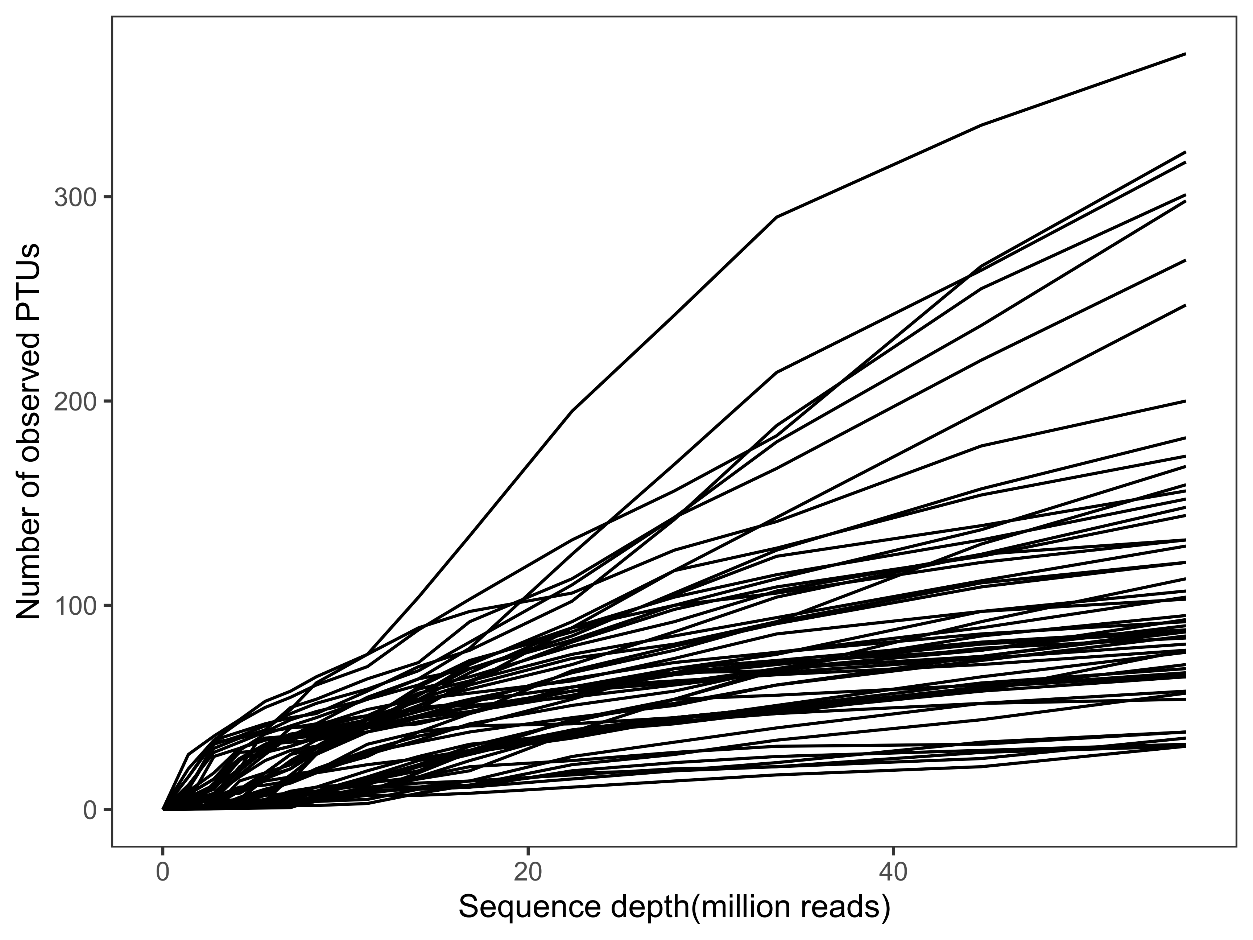


**Fig. S4.** Rarefaction curve of plasmid taxonomic units (PTUs) for different samples. The curve shows the accumulation of observed PTUs with increasing numbers of randomly sub-sampled sequences across all samples.


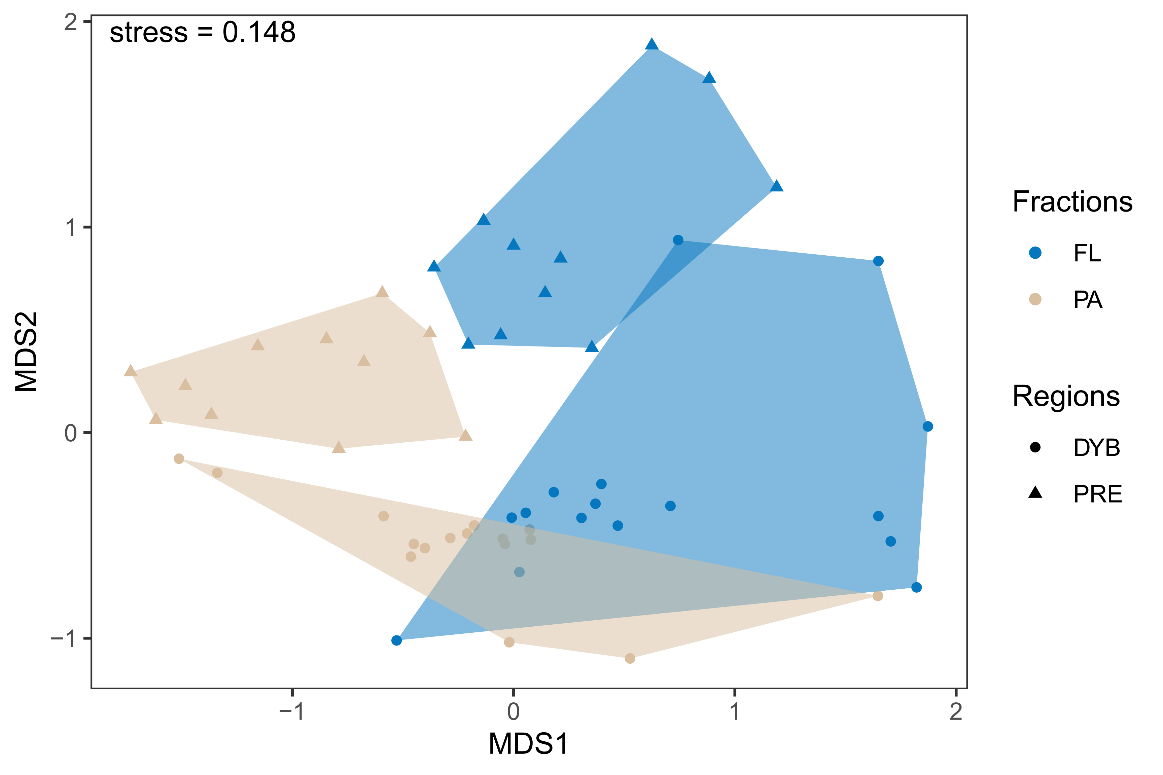


**Fig. S5.** Non-metric multidimensional scaling (NMDS) analysis based on plasmid taxonomic unit (PTU) abundance. NMDS based on Bray-Curtis’s dissimilarity illustrates the differences in plasmid community structure in different regions and different fractions.


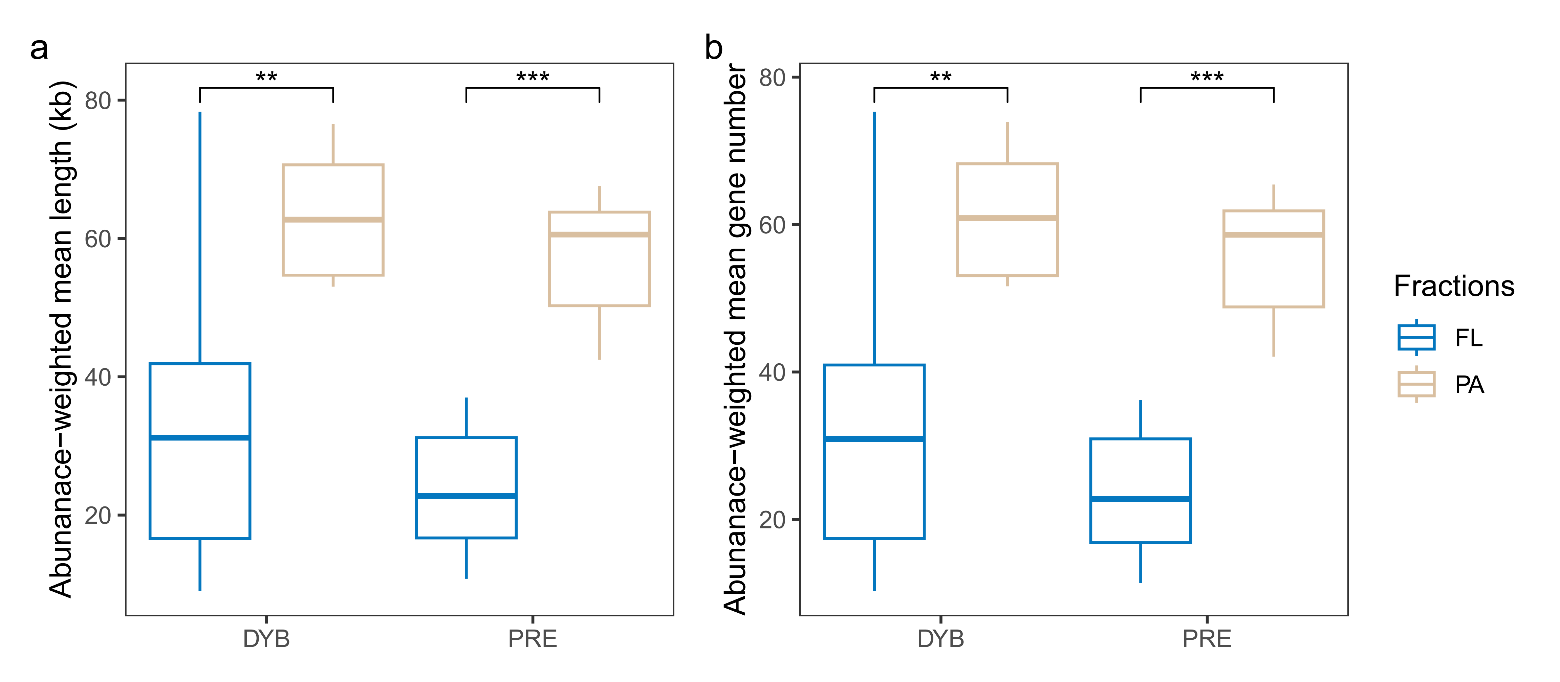


**Fig. S6.** Abundance-weight mean plasmid length and gene number across PA and FL fractions in the PRE and DYB.


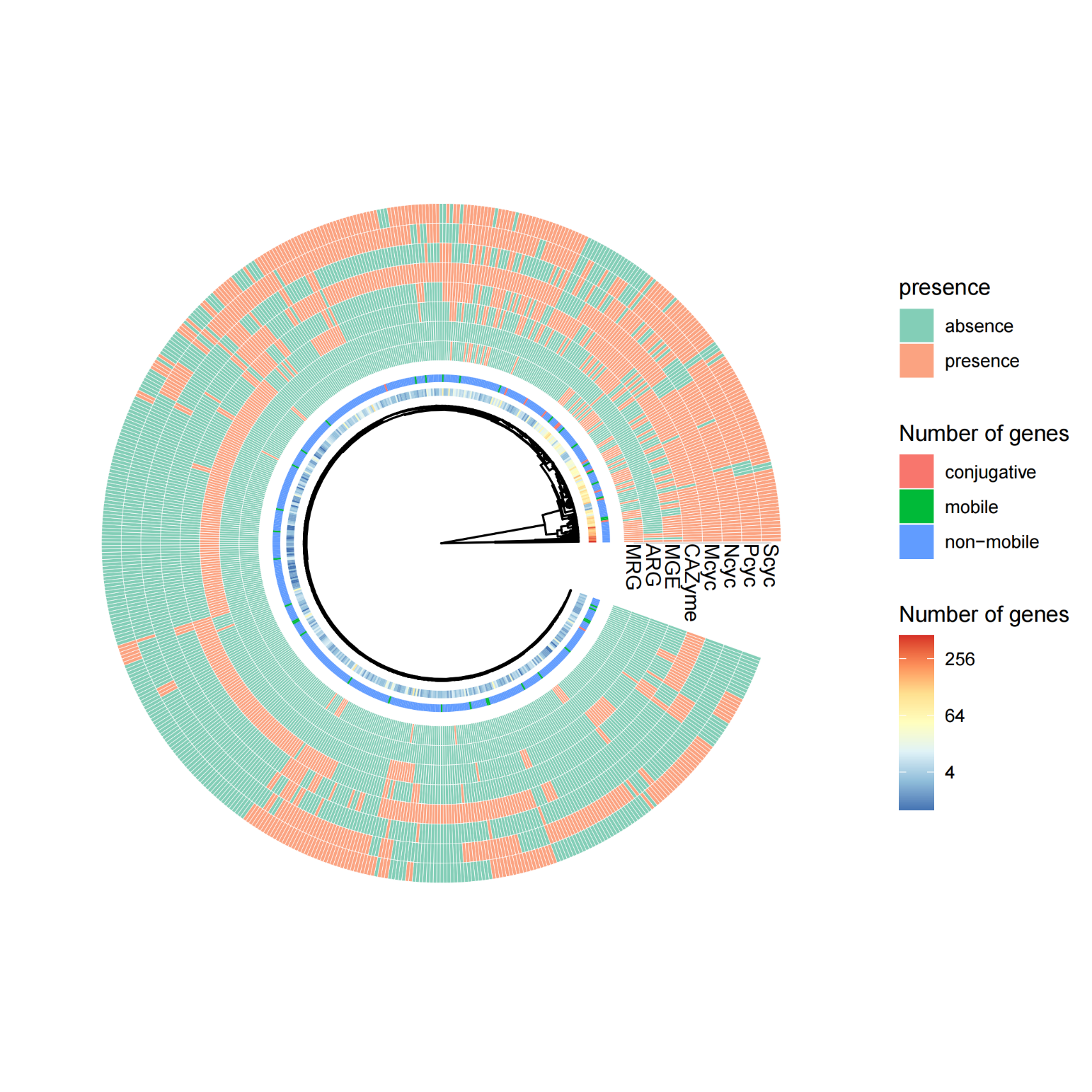


**Fig. S7.** Distribution of auxiliary metabolic genes, resistance genes, and mobile genetic elements across PTUs. Only PTUs containing at least one gene are shown. Clustering of PTUs is based on gene presence-absence patterns. MRG: metal resistance genes; ARG: antibiotic resistance genes; MGE: mobile genetic elements (mainly transposases, integrases, and insertion sequences); CAZyme: carbohydrate-active enzyme; MCcyc: methane cycling genes; Ncyc: Nitrogen cycling genes; Pcyc: Phosphorus cycling genes; Scyc: Sulfur cycling genes.


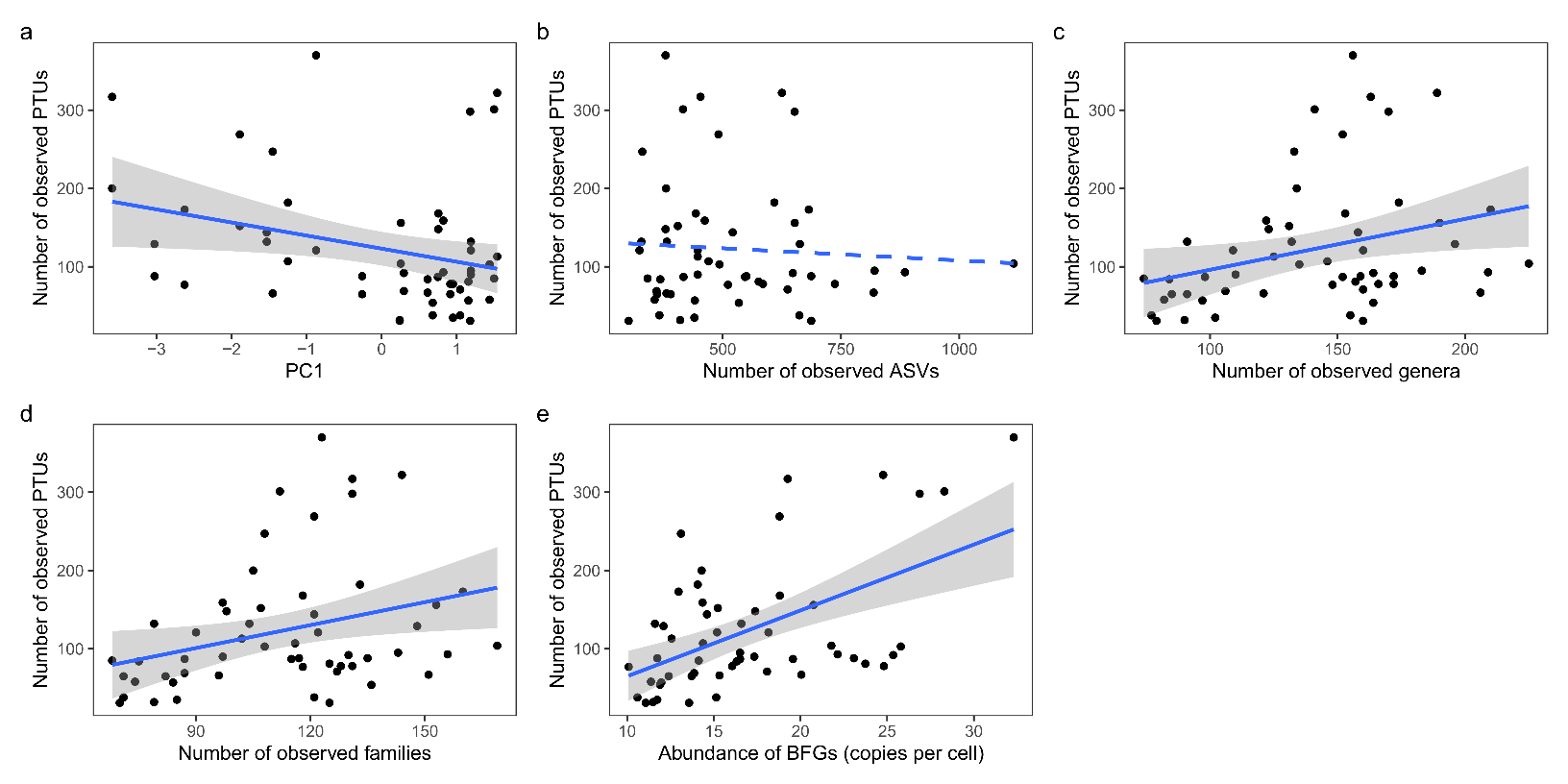


**Fig. S8.** Linear regression relationships between plasmid diversity and environmental gradient, microbial host diversity, and biofilm gene diversity. The figure presents linear regression relationships between plasmid diversity, represented by the number of PTUs, and five key predictors: (a) The principal component of the environmental gradient (PC1);

(b) number of observed ASVs; (c) number of observed genera; (d) number of observed families; (e) abundance of biofilm-forming gene (BFGs) per cell.


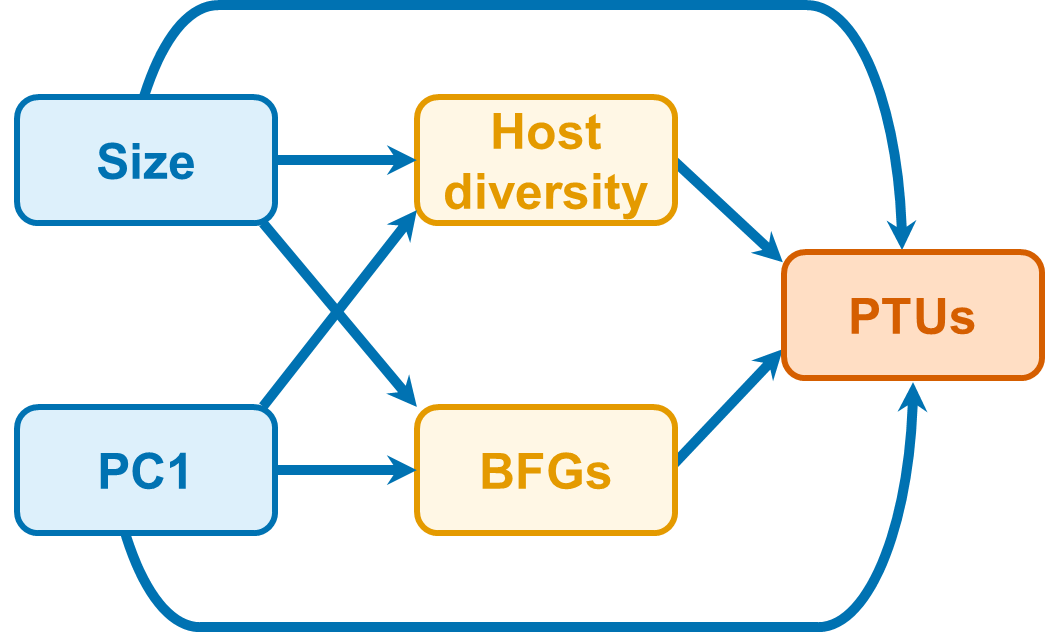


**Fig. S9.** Conceptual path framework illustrating hypothesized effects of environmental gradient and fractions on plasmid diversity. This diagram presents the theoretical hypothesis framework corresponding to the structural equation model in Fig. 4, illustrating the hypothesized causal pathways through which the environmental gradient (represented by PC1) and different size fractions (PA vs. FL) may potentially drive changes in plasmid diversity by influencing host diversity (family level) and the abundance of biofilm-forming genes (BFGs).


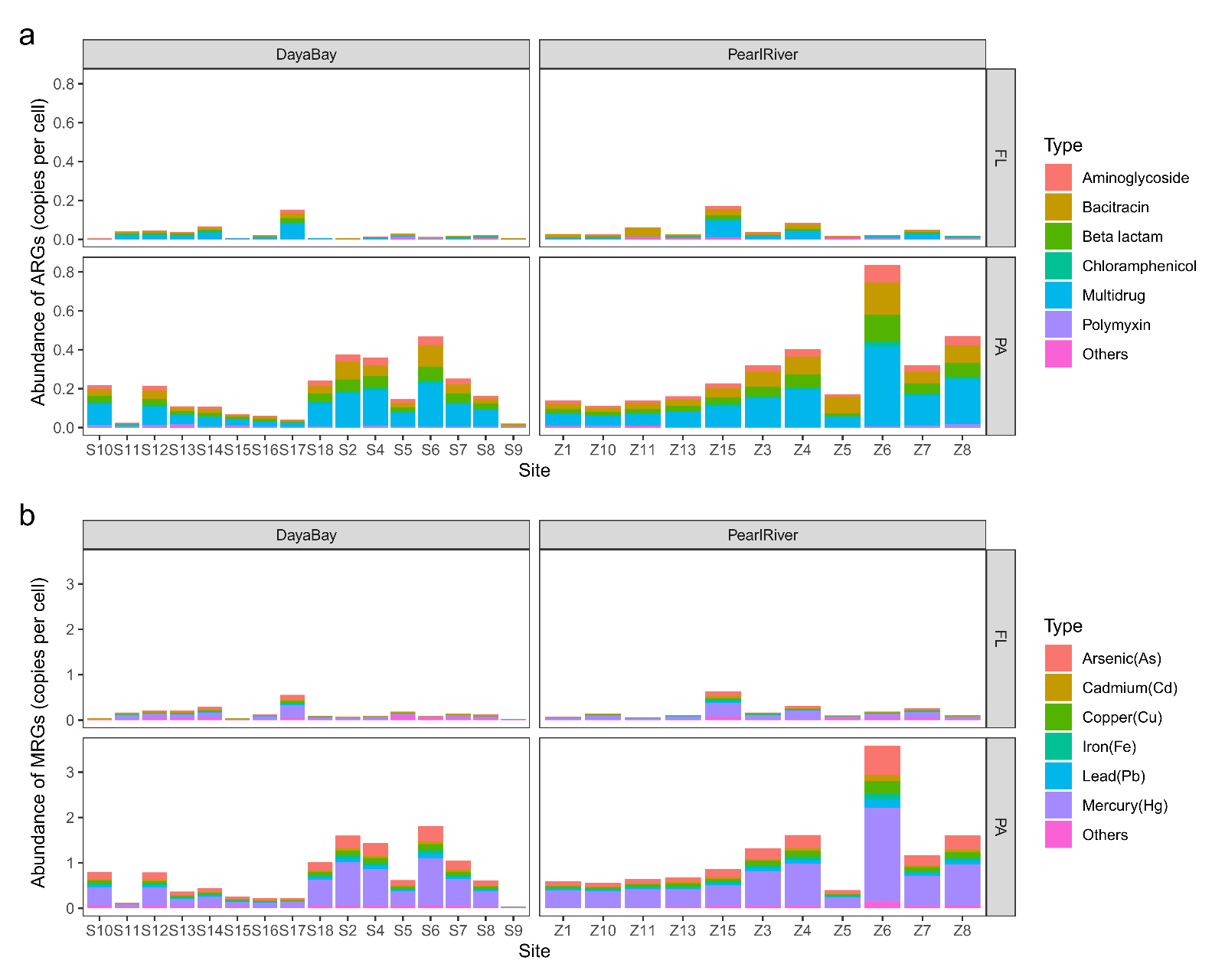


**Fig. S10.** Composition of ARGs and MRGs dominant types across different samples. The normalized abundance of the top 6 types of (a) ARGs and (b) MRG types across all samples.
